# Microstate Dynamics in Working Memory: Exploring Spatial Information Coding of Stimulus and Behavioral Performance

**DOI:** 10.1002/brb3.70765

**Published:** 2025-08-22

**Authors:** Hamideh Norouzi, Mohammad Reza Daliri

**Affiliations:** ^1^ Neuroscience and Neuroengineering Research Lab., Biomedical Engineering Department, School of Electrical Engineering Iran University of Science & Technology (IUST) Tehran Iran

**Keywords:** behavioral performance, cognitive function, EEG microstate, memory‐guided saccade, working memory

## Abstract

Background

EEG microstate analysis provides insights into the spatial and temporal dynamics of brain activity during cognitive tasks. The four canonical microstates (classes A, B, C, and D) have been widely reported and associated with various cognitive functions. However, the relationship between microstate parameters and behavioral responses in cognitive functions, such as working memory (WM), has not been sufficiently investigated. This study investigates how microstate dynamics relate to WM performance during a memory‐guided saccade (MGS) task.

**Methods:**

EEG and Eye‐tracking data were recorded from participants performing an MGS task at two target eccentricities (near and far). Saccade error was used as a behavioral index of WM performance. Microstate parameters (occurrence, coverage, duration, and transition probability) were computed for the four canonical microstates during the trials.

**Results:**

Our analysis revealed a significant reduction in the coverage of microstate C, often associated with the default mode network, during the memory maintenance interval compared to baseline. Moreover, a notable increase was observed in the duration of microstate D, considering polarity during the memory interval, which could be related to the frontoparietal control network (FPCN). Notably, the transition probability (TP) from D+ to D‐ during the memory duration correlated with saccade errors, indicating a behavioral predictive capacity. Furthermore, we identified distinct patterns of microstate D transitions to other microstates that differed significantly between the near and far target conditions, suggesting a functional role in spatial coding.

**Conclusion:**

Microstate dynamics, particularly those of microstate D, play a dual role in spatial WM by supporting information coding and predicting behavioral accuracy. The polarity‐specific transitions within microstate D provide a neural signature of WM performance, with implications for understanding network‐level mechanisms underlying spatial memory and saccade control.

## Graphical abstract

The schema of the microstate analysis procedure is as follows: (a) For each subject and each trial, a time interval of 500 ms before stimulus onset to 1500 ms after stimulus offset was selected for analysis. The black dashed line indicates stimulus onset, and the red dashed line indicates stimulus offset (300 ms). (b) Global field power (GFP) was calculated for each trial. (c and d) Maps at local maxima of GFP were chosen for clustering analysis; the red asterisks indicate the local maximum. (e) Spatial clustering of topographic maps across trials and subjects.

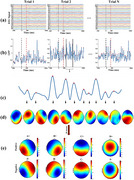

**Abbreviations**
DMN: default mode network
EEG: electroencephalography
FPCN: frontoparietal control network
GFP: global field power
MGS: memory‐guided saccade
TP: transition probability
WM: working memory

## 1 Introduction

Electroencephalography (EEG) is a tool for measuring brain electrical potentials through electrodes placed on the scalp with high temporal resolution. Several techniques have been employed to extract temporal and spatial information from EEG data, one of which is microstate analysis. This method analyzes multichannel EEG recordings to identify a series of patterned, transient, and quasi‐stable brain states (Khanna et al. 2015; Liu et al. 2020). These states are thought to represent the simplest manifestations of human cognitive processes and typically last a few tens of milliseconds. Therefore, microstates are referred to as “the atoms of thought” (Lehmann 1990).

The high temporal resolution of EEG allows for microstate analysis to examine the fundamental building blocks of cognition and dynamic patterns of brain network activity. A variety of studies utilizing classification algorithms have consistently recognized four distinct classes of microstates, designated as Classes A, B, C, and D. These groups of EEG microstates are commonly known in the literature as canonical microstates (Michel and Koenig 2018).

The topography of these four microstate classes exhibits distinct spatial patterns, including an asymmetric orientation: Class A (right frontal to left occipital) and Class B (left frontal to right occipital), and two symmetric topographies: Class C (frontal to occipital direction) and Class D (frontal to occipital direction, but with a greater maximum frontal midline orientation than Class C) (Lehmann et al. 2005; Kindler et al. 2011).

Previous studies indicate that Class A is associated with a phonological processing network, while Class B is related to a visual network. Class C is associated with regions encompassing the prefrontal cortex and posterior cingulate cortex, which are part of the default mode network (DMN). Finally, Class D is linked to the central executive network and the frontoparietal control network (FPCN) (Michel and Koenig 2018; Britz et al. 2010; Custo et al. 2017; Bréchet et al. 2019).

To investigate microstates during a resting state or task‐related activity, several parameters are calculated and analyzed: occurrence (the average frequency of microstate dominance per second throughout the entire recording), duration (the average time a microstate remains stable), coverage (the proportion of the total recording time that the microstate is dominant), and transition probability (TP) (the probability of transitioning from one state to another).

Understanding the functional significance of EEG microstates and their sequences is essential, as they serve as biomarkers for various neurological and psychological disorders, including depressive disorders (Yan et al. 2021; He et al. 2021), autism (Nagabhushan Kalburgi et al. 2020), Alzheimer's disease (Tait et al. 2020; Lassi et al. 2023), Parkinson's disease(Pal et al. 2021; Chu et al. 2020), schizophrenia (K. Kim et al. 2021; Sun et al. 2021), attention deficit hyperactivity disorder (Sen Wu et al. 2024; Luo et al. 2021).

EEG microstates represent the brain's cognitive processing and its responses to external stimuli based on its current state. Recent research indicates that microstates affect perception (Britz et al. 2014) and cognition (Asha et al. 2024), as well as characterize spontaneous thoughts (Milz et al. 2016). This evidence implies that varying distributions of active neuronal populations may fulfill distinct roles, potentially corresponding to different transient functional microstates within the brain.

Working memory (WM) is a cognitive system that allows for the retention of a small amount of information for a short period of time, making it available for other cognitive activities(Chai et al. 2018; Barak and Tsodyks 2014). It can be categorized into three phases: encoding, maintenance, and information retrieval. The effectiveness of WM relies on top‐down regulatory processes, which can be assessed by examining the functional connectivity between prefrontal and parietal regions (Awh and Jonides 2001; Norouzi and Daliri 2024).

There have been limited studies on the role of microstates in analyzing cognitive processes involved in WM, such as increasing the duration and coverage of Class C in emotional WM (Pan et al. 2020) and age‐related modulation of transition dynamics between Classes C and D and their impact on spatial WM performance (Jabès et al. 2021). To investigate the relationships between static microstate parameters and dynamic microstate changes in WM during an N‐back task, Tamano et al. conducted a study that revealed significant changes in event‐related microstate dynamics mainly influenced by different WM loads. In contrast, their study found no significant differences in static microstate parameters (Tamano et al. 2022).

Examining behavioral metrics derived from eye‐tracking data and neurological signals in cognitive tasks is consistently beneficial for neuroscientists and psychologists. These approaches enhance our understanding of the mechanisms underlying eye movements and their neural correlates in cognitive processes (Skaramagkas et al. 2023). One specific focus of this research is the working memory‐guided saccade (MGS) paradigm, which examines eye movements directed by WM content. In the MGS task, subjects are instructed to voluntarily execute saccades toward a target that was previously presented (Norouzi et al. 2021).

In this study, we employed the MGS task, combining simultaneous EEG and eye‐tracking recordings from 17 participants, to examine the following research objectives: (1) We provide evidence of the spatiotemporal dynamics of EEG activity during WM. To examine EEG dynamics, we employed microstate analysis, which involves identifying specific topographic patterns of brain electrical activity. We analyzed the temporal dynamics of microstates during working MGSs (fixation, visual (encoding), and maintenance) using a single‐trial approach that differs from traditional methodological paradigms (Asha et al. 2024; Perrottelli et al. 2024). (2) We measured saccade error as a behavioral metric in the MGS task and examined its relationship with microstate parameters. Specifically, we compared microstate characteristics between groups with high errors (saccade error > 1.5°) and low errors (saccade error < 0.5°). (3) To determine whether microstate analysis contributes to coding spatial information, we compared microstate parameters under two visual angle conditions (6° and 12°).

## 2 Materials and Methods

### 2.1 Participants

Seventeen healthy individuals (10 women and seven men), aged 19–29 years, were recruited for this study. The experiment comprised 120 trials, divided into three sessions of 40 trials each. The ethical guidelines outlined in the 1964 Declaration of Helsinki and its later revisions were followed during this experiment.

### 2.2 Recording Setup

Subjects were positioned 60 cm from the task monitor (19 in., resolution:1366 × 768 pixels, refresh rate: 60 Hz), and their heads were stabilized using a chinrest. Eye movement data were recorded with an eye tracker (SR Research, EyeLink 1000 plus) in the monocular (right eye) Pupil‐CR recording mode at 1000 Hz. It is important to note that the eye‐tracking device was calibrated with a standard nine‐point monocular calibration before each session to ensure precise measurement of eye movement characteristics. The participants' EEG signals were simultaneously recorded using 20 electrodes in accordance with the 10–20 system. The electrode placements included: FP1/2, F7/8, F3/4, Fz, T7/8, C3/4, Cz, P7/8, P3/4, Pz, O1/2, and Oz.

### 2.3 Behavioral Task

**FIGURE 1:**
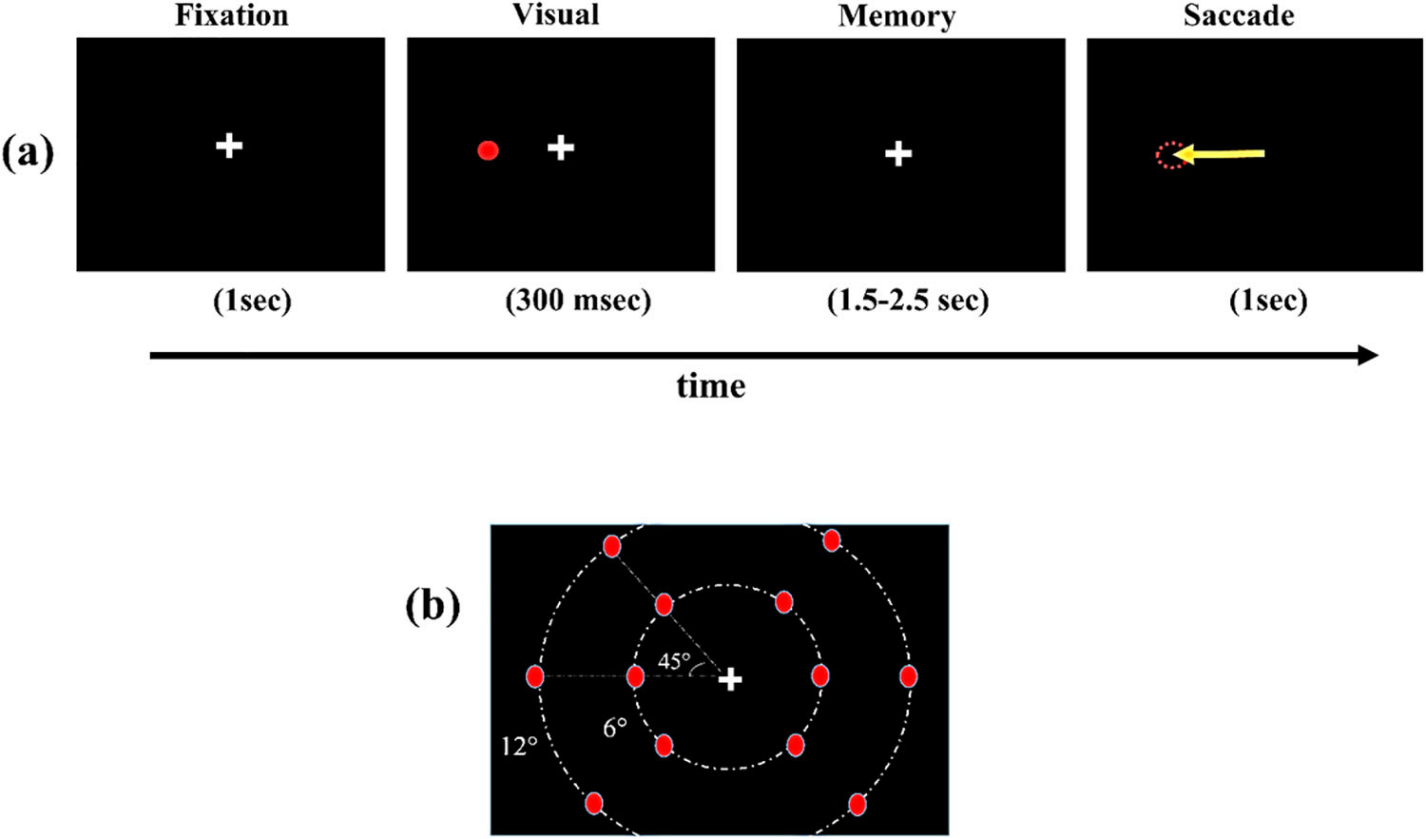
Experimental paradigm of memory‐guided saccade task. (a) At the beginning of each trial, the subject fixated on the fixation cross in the center of the screen. A red stimulus was displayed in the visual duration. Then, the stimulus disappeared, and the subject continued to fixate during the memory period. After that, the white fixation cross was removed, and the subject had to make a saccade to the remembered stimulus location. (b) Twelve possible locations for stimulus presentation.

In this experiment, we employed a MGS task. Participants were instructed to memorize the location of a presented stimulus. Then, they executed a saccade to the memorized location, as depicted in Figure 1a. According to this figure, each trial consists of four steps. In the first step (fixation), the fixation cross was displayed in the center of the screen for 1 s. In the second step (visual), a red stimulus (0.5° in diameter) was presented as a cue for 300 ms. In the third step (memory), the stimulus was removed, and the subject retained the location of the stimulus for 1.5–2.5 s. In the last step, the subject had 1 s to execute a saccade to the stimulus location. The stimuli were presented at visual angles of 12° or 6° (Figure 1b), randomly selected from 12 possible locations. Each stimulus was shown 10 times, resulting in a total of 120 trials.

### 2.4 Eye‐Tracking Data Analysis

In this experiment, we utilized eye‐tracking data analysis to verify trial accuracy. To ensure accurate data collection, participants were instructed at the experiment's outset to minimize blinking during the trial, although they were allowed to blink and rest their eyes between trials. After the experiment, trials that included blinking were removed from the analysis. Additionally, trials were eliminated if participants incorrectly fixated during the fixation, visual, and memory intervals (Figure 1a) or if saccadic errors exceeded 2° during the saccade phase. Saccade error, calculated using Equation (1), was defined as the Euclidean distance (in degrees of visual angle) between the target location and the saccade landing point. This distance was determined from the *x* and *y* coordinates (in pixels) of both the target location and the saccade landing point.

(1) $$\mathrm{Saccade}\;\mathrm{Error}\left(\mathrm{dva}\right)=\frac{\sqrt{{\left(x_{\mathrm{target}\;\mathrm{location}}-x_{\mathrm{saccade}\;\mathrm{landing}\;\mathrm{point}}\right)}^{2}+{\left(y_{\mathrm{target}\;\mathrm{location}}-y_{\mathrm{saccade}\;\mathrm{landing}\;\mathrm{point}}\right)}^{2}}}{\mathrm{Pixel}\;\mathrm{per}\;\mathrm{degree}}$$

### 2.5 EEG Preprocessing

EEG analysis was performed in MATLAB. Initially, each EEG session was filtered using a sixth‐order Butterworth filter (1–30 Hz). Following this, all data were re‐referenced to the common average reference.

EEG data are known to differ across various recording sessions. Furthermore, variations in the recording environment and individual anatomical differences can lead to distinct EEG patterns among participants. In this study, we utilized the *z*‐score method (Hu and Zhang 2019) for normalization, which transforms values from a normal distribution into a standard normal distribution. We conducted our analysis on the data from correct trials after the normalization of the signals.

### 2.6 Microstate Analysis

**FIGURE 2:**
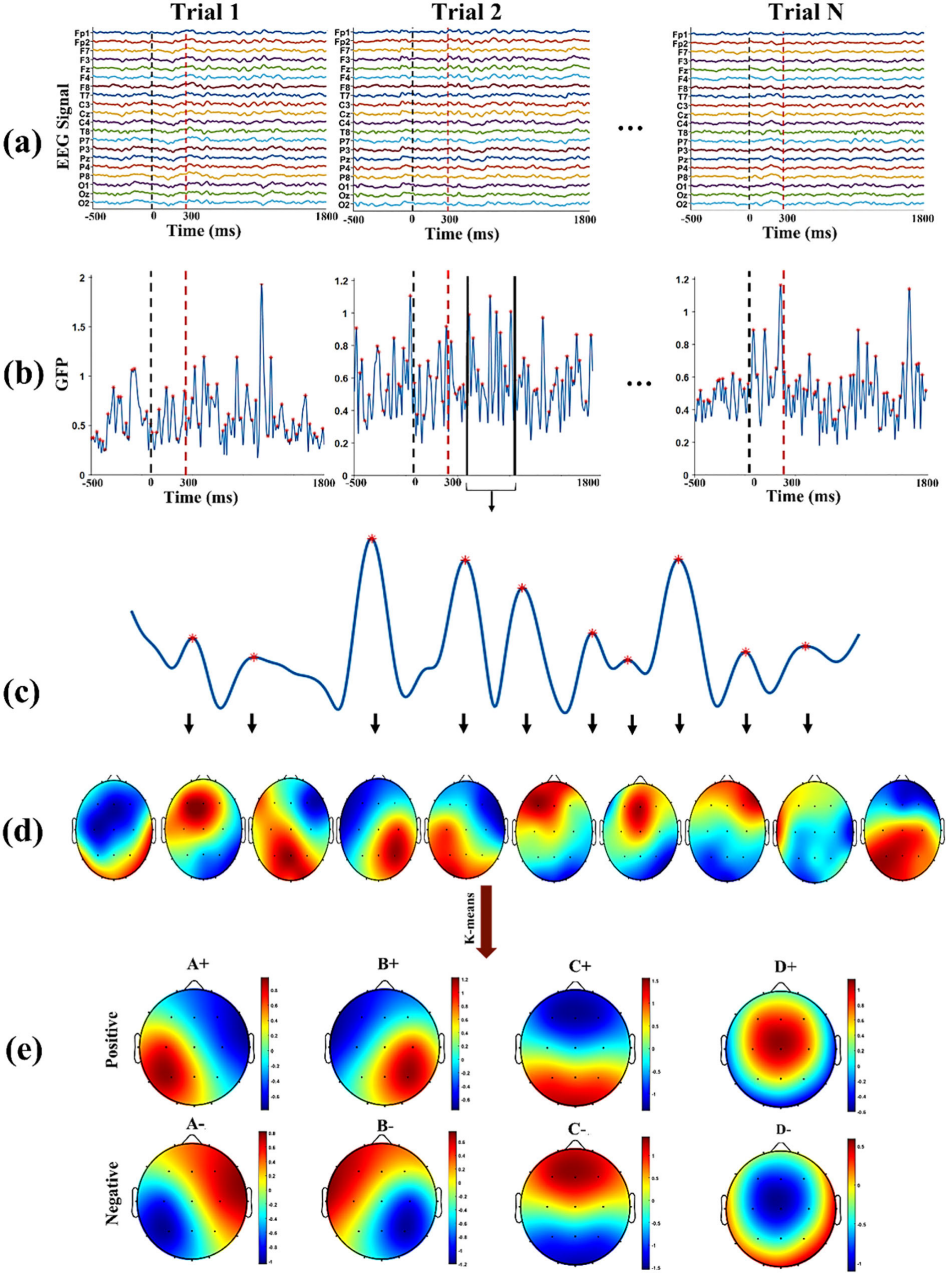
The schema of the microstate analysis procedure. (a) For each subject and each trial, a time interval of 500 ms before stimulus onset to 1500 ms after stimulus offset was selected for analysis. The black dashed line indicates stimulus onset, and the red dashed line indicates stimulus offset (300 ms). (b) Global field power (GFP) was calculated for each trial. (c and d) Maps at local maxima of GFP were chosen for clustering analysis; the red asterisks indicate the local maximum. (e) Spatial clustering of topographic maps across trials and subjects.

Microstate analysis aims to identify the most representative maps in fixation, visual, and memory time intervals. For this study, we considered the interval from 500 ms before the presentation of the stimulus to 1.5 s after the removal of the stimulus as the period for trial analysis (Figure 2a).

To investigate the microstates during the trial, we first calculated the global field power (GFP), which represents the standard deviation of EEG signals across electrodes for each trial (Figure 2b). Time points exhibiting GFP maxima were identified, as they indicate a relatively high signal‐to‐noise ratio (Poulsen et al. 2018). Consequently, we extracted the spatial map corresponding to the maximum GFP values for each trial (Figure 2c). After compiling the maps from all trials and subjects, we removed samples with outlier values. We then employed the *k*‐means algorithm to categorize the remaining maps into eight distinct clusters. According to Figure 2e, these eight clusters were classified into four main clusters regardless of polarity (A, B, C, and D). According to the labels used in previous research, microstate A has a right frontal to left occipital orientation. Microstate B has a left frontal to right occipital orientation. Microstate C has a frontal to occipital orientation, and microstate D has a midline frontal topography(Pan et al. 2020; Khanna et al. 2015).

After clustering and obtaining microstate prototypes, in the back‐fitting step, each of the EEG signal samples is assigned to microstate labels based on the similarity value that corresponds to the spatial correlation criterion computed as follows (Poulsen et al. 2018; Mheich et al. 2015):

(2) $$\mathrm{Cor}r^{k}\left(n\right)=\frac{\sum_{i=1}^{C}Xn_{i}\cdot ak_{i}}{\sqrt{\sum_{i=1}^{C}Xn_{i}^{2}}\cdot \sqrt{\sum_{i=1}^{C}ak_{i}^{2}}}$$

where Corr is spatial correlation between *Xn* and *ak*, *Xn* is the *n*th time sample of the recorded EEG, *ak* is the prototypical map for the *k*th microstate cluster, *K* is the number of clusters (microstate classes), and *C* is the number of EEG channels.

It is well known that EEG recordings contain a considerable amount of unwanted noise. This noise can lead to the emergence of brief microstate segments during clustering or back‐fitting. To mitigate this problem, temporal smoothing can be employed, where an EEG sample is classified into a microstate category not only on its topographical similarity to the microstate prototype, but also by considering the microstate labels of the preceding and following samples. In our study, we implemented a temporal smoothing time window of 30 ms (Tamano et al. 2022). After back‐fitting and temporal smoothing, we obtained a sequence of microstate labels for each trial. Subsequently, for each trial, microstate parameters including occurrence, duration, coverage, and transmission probability were calculated.

Occurrence refers to the average frequency at which a microstate becomes dominant. Time coverage represents the percentage of the analysis period covered by a specific microstate. Duration is the average length of time that a microstate has been continuously present. Transition probabilities between microstate classes are calculated as another parameter for microstate analysis (Poulsen et al. 2018).

### 2.7 Statistical Analysis

To investigate behavioral performance between two groups with different spatial positions (far and near eccentricity), we conducted a Wilcoxon signed‐rank test between subjects. For the EEG microstate analysis over time, we employed a two‐way analysis of variance (ANOVA) with Tukey's multiple comparison test, considering the factors of microstate class (A, B, C, and D) and time interval (fixation, visual, and memory). In addition, to investigate changes in the microstate parameters while considering polarity during the memory duration, we utilized a one‐way ANOVA with Tukey's multiple comparison test. To assess the relationship between microstate parameters and behavioral performance, we applied Pearson correlation analysis.

## 3 Results

### 3.1 Behavioral Results

In the behavioral analysis, each participant's correct trials (*n* = 87 ± 14) were classified as low error (saccade error < 0.5°) or high error (saccade error > 1.5°). A signed‐rank test on participants' average saccade errors within each group revealed a significant difference between low and high error conditions (*p* < 0.001).

In another aspect of the behavioral analysis, as illustrated in Figure 1b, participants making saccades to randomly selected targets. Previous studies have suggested that cognitive processes associated with WM can influence saccade characteristics (Hutton 2008). To explore this, we compared average saccade errors in near and far eccentricity conditions using a signed‐rank test. This revealed significantly greater saccade errors in the far condition (*p* < 0.01).

### 3.2 Comparison of Microstate Parameters Between Fixation, Visual, and Memory Time Intervals

In this study, we computed microstate parameters including occurrence, coverage, and duration separately for each trial across three distinct periods: the fixation period (500 ms before stimulus presentation), the stimulus presentation period (300 ms), and the memory period (1.5 s). Subsequently, we calculated the average of these parameters for each subject. The values of the microstate parameters (occurrence, duration, and coverage) for each class (A, B, C, and D) and for each time interval (fixation, visual, and memory) are presented in Table 1.

**TABLE 1:** Microstate parameters in different phases of the MGS task.

State	Fixation			Visual			Memory		
	Occurrence (N/s)	Coverage	Duration (ms)	Occurrence (N/s)	Coverage	Duration (ms)	Occurrence (N/s)	Coverage	Duration (ms)
A	5.81 ± 0.17	0.24 ± 0.01	40.68 ± 1.08	6.12 ± 0.16	0.23 ± 0.01	35.50 ± 0.98	5.68 ± 0.12	0.24 ± 0.01	42.34 ± 0.65
B	5.81 ± 0.14	0.24 ± 0.01	40.62 ± 1.05	6.38 ± 0.16	0.25 ± 0.01	37.18 ± 1.04	5.98 ± 0.07	0.26 ± 0.01	43.03 ± 0.64
C	6.06 ± 0.14	0.27 ± 0.01	43.83 ± 1.10	6.43 ± 0.18	0.26 ± 0.01	37.98 ± 1.35	5.76 ± 0.15	0.25 ± 0.01	43.06 ± 0.60
D	5.83 ± 0.24	0.24 ± 0.01	41.38 ± 1.10	6.44 ± 0.32	0.25 ± 0.02	36.52 ± 1.53	5.73 ± 0.23	0.25 ± 0.02	43.61 ± 1.13

*Note*: Mean and SEM values of microstate parameters (occurrence, duration, and coverage) for each class (A, B, C, and D), for each condition (fixation, visual, memory).

Abbreviation: SEM, standard error of the mean.

**FIGURE 3:**
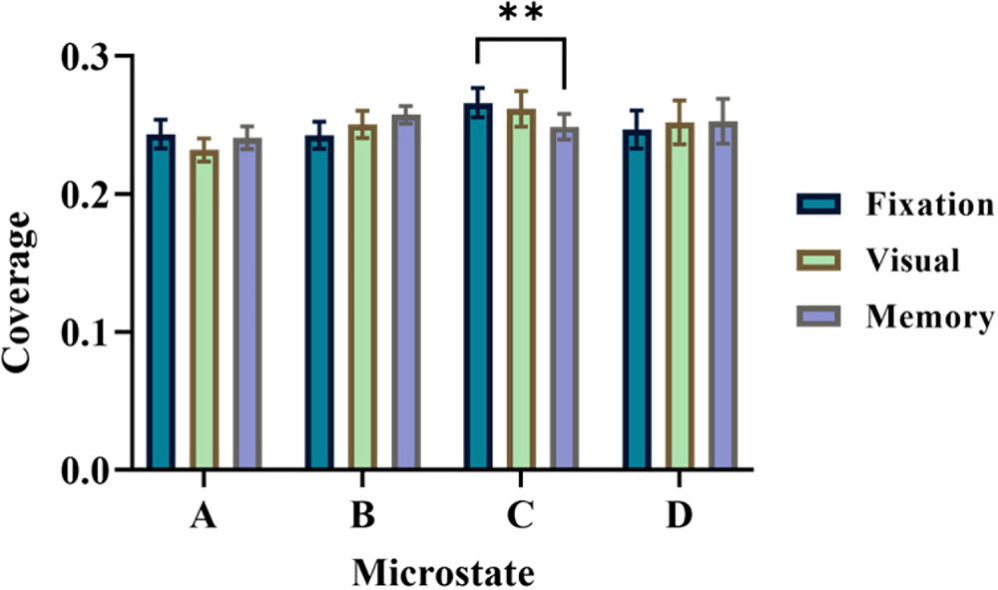
Comparison of microstate parameter (coverage) between fixation, visual, and memory duration. Data are mean ± SEM (two‐way ANOVA with Tukey's multiple comparison test, **p < 0.01).

To examine changes in microstate parameters during trials, we conducted a two‐way ANOVA with Tukey's multiple comparison test between subjects, with factors of time interval (including three periods: fixation, visual, and memory) and microstate class (including four main classes: A, B, C, and D). According to Table 2, the results of the two‐way ANOVA analysis for the coverage parameter show that the interaction between the main factor of the microstate and the main factor of the time interval is significant (*F* (6,128) = 3.47, *p* = 0.003). Tukey's multiple comparison test result in Figure 3 indicates that the coverage of microstate C has a significant difference between fixation and memory intervals (*p* = 0.004). As shown in Figure 1a, during both the fixation and memory periods, participants were required to look at a fixed point in the center of the screen. The key distinction between the two periods lies in the memory period, during which subjects were required to actively maintain the location of the displayed stimulus. Accordingly, the greater amount of microstate C coverage during the fixation period compared to the memory period is consistent with findings from previous studies, which suggest that Microstate C is associated with brain regions associated with the default mode system. Notably, these studies reported a decrease in microstate C coverage during task engagement (Seitzman et al. 2017).

**TABLE 2:** Statistical comparison of coverage parameter during the MGS task.

Factor	*F*‐value	*p*‐value
Microstate	*F* (3, 64) = 0.58	0.62
Condition	*F* (1.96, 125) = 0.05	0.94
Microstate × condition	*F* (6, 128) = 3.47	0.003**

*Note*: Result of two‐way ANOVA for coverage parameter with factors: microstate (A, B, C, D) and condition (fixation, visual, memory).

**p < 0.01).

### 3.3 Comparison of Microstate Parameters in Memory Duration

The primary objective of this study is to investigate brain microstates during the memory period, specifically their relation to information retention and manipulation. To achieve this, we analyzed microstate parameters throughout the memory interval, employing a novel approach that includes the consideration of polarity (positive/negative) (see Figure 2e). To account for polarity, we identified the four clusters labeled as positive in Figure 2e as the main microstates. Topographic maps exhibiting a positive correlation with these were labeled positively (A+, B+, C+, and D+), while those with a negative correlation were labeled negatively (A−, B−, C−, and D−).

**FIGURE 4:**
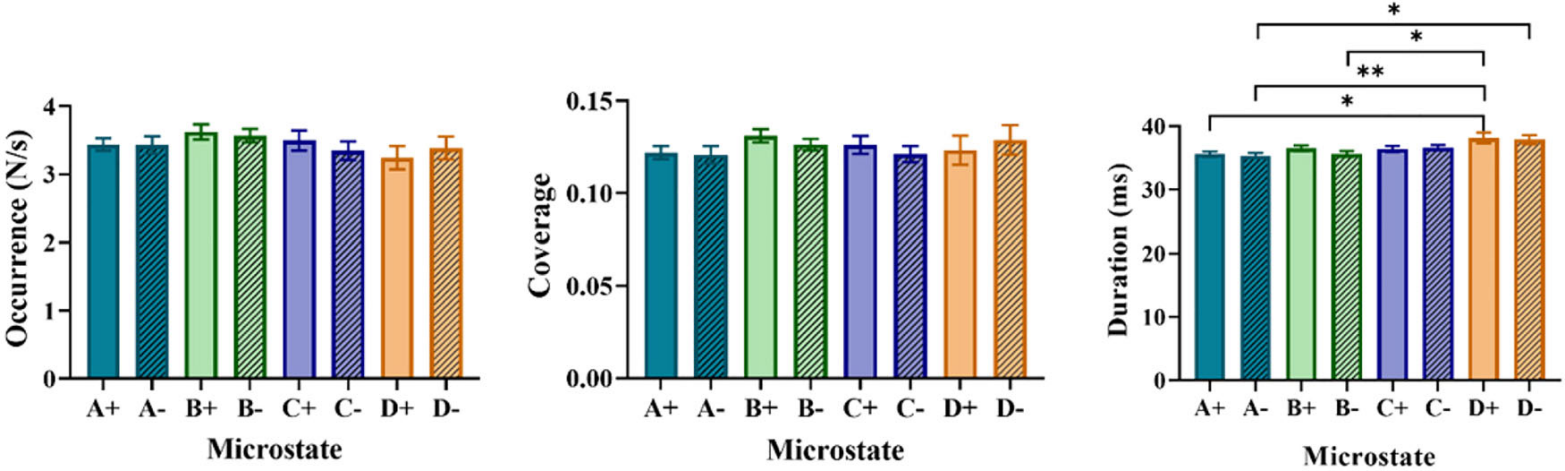
Comparison of microstate parameters considering polarities (positive/negative) in memory duration. Bar plots show the mean of the microstate parameters (left, occurrence; middle, coverage; right, duration) for memory duration. Data are mean ± SEM (significant *p*‐values from one‐way ANOVA with Tukey's multiple comparison test are indicated. *p < 0.05, **p < 0.01).

To examine microstate parameters over the duration of memory, results across all trials were averaged for each subject and one‐way ANOVA with Tukey's multiple comparison test between subjects was used. As illustrated in Figure 4, the durations of Classes D+ and D− showed a significant increase compared to other microstate classes, potentially highlighting the role of Class D in information retention during WM tasks.

### 3.4 Relationship Between Microstate Parameters and Saccade Error

**FIGURE 5:**
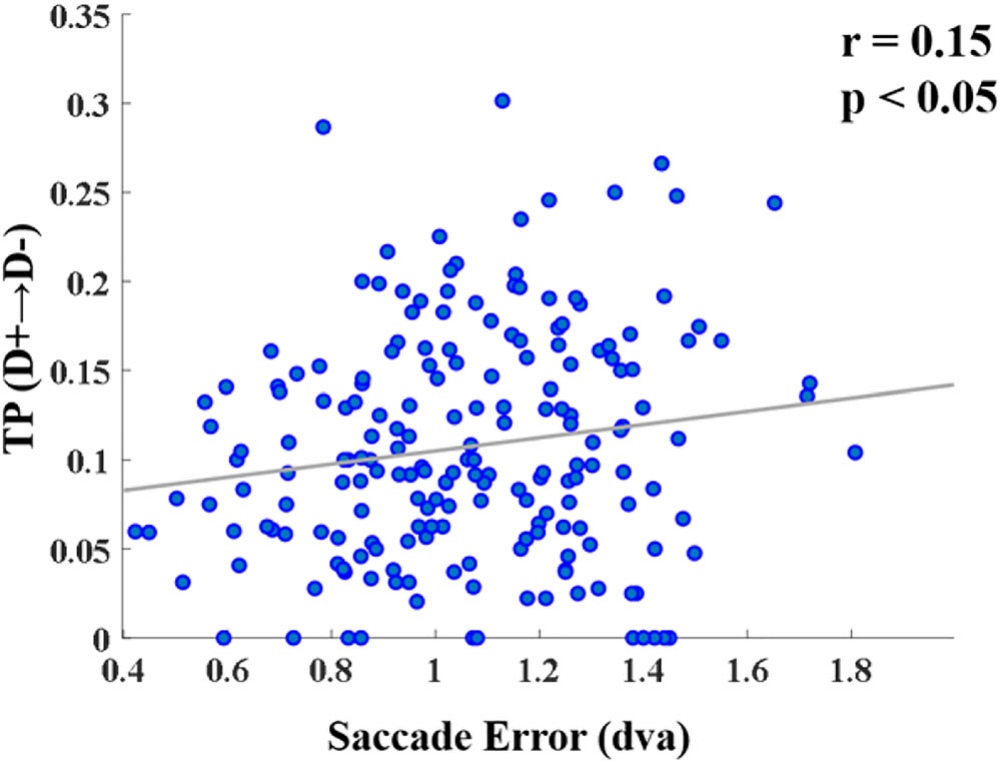
Pearson correlation between saccade error and transition probability (D+→D‐) in the maintenance interval.

We computed the Pearson correlation coefficient between saccade errors and microstate parameters during the maintenance period to assess the relationship between behavioral performance in the MGS task and microstate parameters. We averaged saccade errors for each of the 12 target positions shown in Figure 1b. Similarly, microstate parameters were computed for the maintenance duration of these trials and subsequently averaged. This approach generated a set of 12 saccade errors and 12 corresponding microstate parameters for each participant. Figure 5 demonstrates a significant positive correlation (*n* = 195, *r* = 0.15, *p* = 0.03) between saccade error and the TP from D+ to D− in the maintenance interval.

### 3.5 Comparison of Microstate Parameters for Low‐Error and High‐Error Groups

**FIGURE 6:**
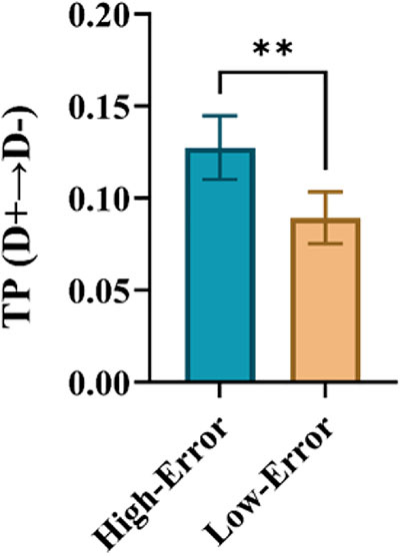
Comparison of transition probability (TP) from D+ to D− in two groups of low error and high error (signed‐rank test, **p < 0.01). Data are mean ± SEM.

As indicated in the behavioral results section, trials were categorized into two distinct groups based on saccade error: low error (saccade error < 0.5°) and high error (saccade error > 1.5°). Given the correlation between saccade error and microstate parameters shown in the previous section, we proceeded to investigate the differences in microstate parameters between the two groups. To do this, we averaged the microstate parameters for each subject during the maintenance interval and conducted a signed‐rank test to evaluate significant differences between the groups across participants. Figure 6 shows that the TP from D+ to D− is significantly greater in the high error group compared to the low error group (*p* = 0.003).

### 3.6 Comparison of Microstate Parameters for Near and Far Condition

**FIGURE 7:**
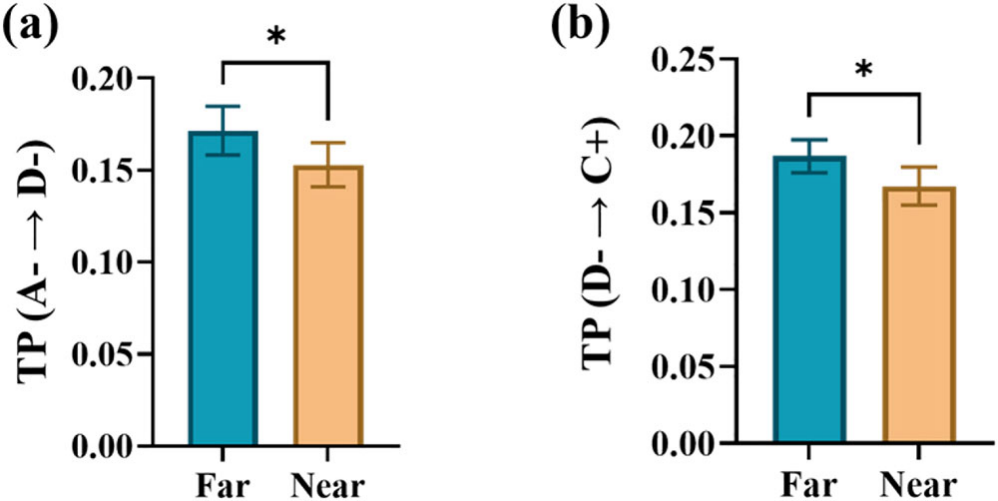
Comparison of microstate parameters between near and far conditions. (a) transition probability (TP) from A− to D− and (b) TP from D− to C+ in two conditions of far and near eccentricity (signed‐rank test, *p < 0.05). Data are mean ± SEM.

The MGS task depicted in Figure 1b divides stimuli into two spatial groups: near (6°) and far (12°). According to Section 3.1, the behavioral analysis reveals significant distinctions between these conditions. To explore the potential role of microstate analysis in coding spatial information during a spatial WM task, we calculated microstate parameters for both the near and far conditions across the memory interval. We averaged these parameters for each participant and employed a signed‐rank test to identify which parameters effectively distinguished between the groups. As illustrated in Figure 7a, the results revealed a significant difference in TP (A− → D−) between the two conditions (*p* < 0.05). Additionally, Figure 7b shows that TP (D− → C+) was significantly different between the groups (*p* < 0.05).

## 4 Discussion

This research aimed to examine the dynamics of microstates and their relationship to behavioral performance during a WM task. To achieve this, a working MGS task was developed, as illustrated in Figure 1a. In each trial, participants were asked to memorize the location of a stimulus presented during a delay interval and then make a saccade to that location in the last second of the trial. The task featured two conditions based on the eccentricity of the presented stimulus: near (6°) and far (12°) (Figure 1b).

To explore the neural correlates associated with the task, we performed a microstate analysis on EEG data obtained from trials across all participants. This analysis identified four distinct topographic maps, labeled A, B, C, and D, as illustrated in Figure 2. Notably, the topographical maps of these four classes were consistent with findings from previous microstate studies (Khanna et al. 2015; Michel and Koenig 2018; Lehmann et al. 2005; Kindler et al. 2011).

In the initial section of our EEG signal analysis results, we assessed parameters from the microstate analysis, such as duration, coverage, and occurrence, across the fixation, visual, and memory intervals. The findings revealed a significant difference in the coverage of Class C between the fixation and memory time intervals. As shown in Figure 3, the coverage of Class C is greater during the fixation period compared to the memory‐delay period.

Numerous studies indicate that microstate Class C is a dominant microstate class in the resting state. It has been shown to be highly sensitive to task manipulation and its coverage is reduced during task conditions compared to rest (Seitzman et al. 2017; Poskanzer et al. 2021). In the present study, the reduced coverage of Class C in the memory duration could be consistent with previous findings that Class C is associated with regions that are part of default mode systems, such as the anterior cingulate cortex, bilateral inferior frontal gyrus, and insula. Activity in these regions is consistent with the DMN (Pan et al. 2020; Panda et al. 2016).

In the second part of our microstate analysis, we aimed to explore the microstates associated with cortical activation during the memory interval, particularly in terms of the maintenance of spatial information. Unlike traditional approaches, we considered the importance of polarity in spatial maps for interpreting electrical brain signals. Consequently, we calculated microstate parameters by incorporating the polarities of the microstates.

Our results indicate that the durations of the D+ and D− microstates during the memory interval were significantly longer than those of other microstates (Figure 4). This suggests that microstate D plays a critical role in cognitive processes such as WM, especially in retaining spatial information. Previous studies have linked microstate D to the central executive network and the FPCN, which encompasses the frontal and parietal regions (Bréchet et al. 2019).

Additionally, we explored the relationship between microstate parameters and saccadic errors as behavioral responses. Our results showed a significant correlation between the TP from D+ to D− and the saccade error rate. As shown in Figure 5, we found that an increase in TP (D+ → D−) during the memory interval was associated with a corresponding increase in saccade error rate. In our behavioral analysis, we categorized the trials into two groups: low error and high error, according to saccade error. We found that during the memory interval, TP (D+ → D−) in the high error group significantly increased compared to that in the low error group (Figure 6), highlighting a correlation between TP (D+→D−) and saccade error. These findings suggest that TP (D+ → D−) during the memory period can serve as a predictor for the saccade error, reflecting behavioral performance in the MGS task.

The increased duration of microstate D during the maintenance interval further supports its involvement in FPCN (Ptak et al. 2017). The correlation between TP (D+→D−) and behavioral performance during the memory phase aligns with previous research pointing to the FPCN's essential role in sustaining attention in WM. It also facilitates the retention of stored information while suppressing task‐irrelevant thoughts during memory‐delay periods (H. Kim 2019; Eryilmaz et al. 2020).

In addition, we compared these parameters based on the location of stimuli (near and far conditions). As mentioned in Section 3.1, our analysis revealed significant differences in behavioral parameters (saccade error), with the near condition exhibiting notably lower saccade errors compared to the far condition. Furthermore, our findings indicated significant differences between TP (A−→D−) and TP (D−→C+) during the memory period of the MGS task under both conditions (see Figure 7). These results suggest that microstate analysis during maintenance interval in the MGS task can predict saccade error as a measure of memory performance, and is also linked to the neural representation of spatial information in WM.

While our study provides valuable insights into the temporal dynamics of brain networks during WM tasks, several limitations should be acknowledged. The relatively small number of participants may limit the generalizability of our findings. Additionally, the absence of multimodal imaging techniques constrains our understanding of the underlying neurobiological mechanisms. Future research could expand on this framework by applying microstate analysis to clinical populations, particularly individuals with psychiatric disorders, where alterations in large‐scale brain network dynamics and WM deficits are commonly reported. EEG microstates offer a window into brain temporal dynamics via brief, quasi‐stable electrical topographies that can be characterized using a variety of metrics; these metrics may contain specific patterns associated with clinical features, and approaches such as machine learning could enhance the detection and interpretation of these patterns (Pacchioni et al. 2025). Incorporating these approaches in future studies could help uncover specific neural signatures related to cognitive dysfunctions and aid in developing precise diagnostic tools for psychiatric disorders.

## 5 Conclusion

To examine the correlation between EEG microstates and behavioral performance in WM, we designed a MGS task. In this task, we quantified saccade error as an indicator of memory performance for each trial. We then analyzed the microstate parameters corresponding to the four canonical microstates (Classes A, B, C, and D) to understand their role in WM processes. Our findings showed a significant decrease in the coverage of microstate C and a notable increase in the duration of microstate D with considered polarity during the memory interval. Furthermore, we found that the TP from D+ to D− during the memory duration was associated with saccade errors and was greater for trials with larger saccade errors than for trials with smaller errors. We also analyzed microstate parameters according to stimulus location (near vs. far conditions), which revealed significant differences during the memory interval. These results suggest that microstate parameters not only predict behavioral performance but also play an essential role in coding spatial information in WM.

## Author Contributions

**Hamideh Norouzi**: methodology, software, writing – original draft, writing – review and editing. **Mohammad Reza Daliri**: conceptualization, supervision, resources, methodology, writing – review and editing.

## Conflicts of Interest

The authors declare no conflicts of interest.

## Peer Review

The peer review history for this article is available at https://publons.com/publon/10.1002/brb3.70765.

## Data Availability

The data can be accessed through reasonable request from corresponding author.
